# Supplementary material for: Probability of sepsis after infection consultations in primary care in the United Kingdom in 2002–2017: Population-based cohort study and decision analytic model
Source: PLoS Med. 2020 Jul 23;17(7):e1003202. doi: 10.1371/journal.pmed.1003202 (PMC7377386; doi:10.1371/journal.pmed.1003202)
Supplement: S1 STROBE Checklist — STROBE, Strengthening the Reporting of Observational Studies in Epidemiology. (DOC) [file pmed.1003202.s001.doc]

S1 STROBE Checklist: Items that should be included in reports of ***cohort studies***

|  | Item No | Recommendation |
| --- | --- | --- |
| **Title and abstract** | 1 | (*a*) Indicate the study’s design with a commonly used term in the title or the abstract  **Title and Abstract** |
|  |  | (*b*) Provide in the abstract an informative and balanced summary of what was done and what was found **Abstract** |
| Introduction | | |
| Background/rationale | 2 | Explain the scientific background and rationale for the investigation being reported **Introduction** |
| Objectives | 3 | State specific objectives, including any prespecified hypotheses **Introduction, last paragraph** |
| Methods | | |
| Study design | 4 | Present key elements of study design early in the paper **Methods, first paragraph** |
| Setting | 5 | Describe the setting, locations, and relevant dates, including periods of recruitment, exposure, follow-up, and data collection **Methods, first paragraph** |
| Participants | 6 | (*a*) Give the eligibility criteria, and the sources and methods of selection of participants. Describe methods of follow-up **Methods, second paragraph** |
|  |  | (*b*) For matched studies, give matching criteria and number of exposed and unexposed **Not applicable** |
| Variables | 7 | Clearly define all outcomes, exposures, predictors, potential confounders, and effect modifiers. Give diagnostic criteria, if applicable **Methods, 2^nd^, 3^rd^ and 4^th^ paragraphs** |
| Data sources/ measurement | 8* | For each variable of interest, give sources of data and details of methods of assessment (measurement). Describe comparability of assessment methods if there is more than one group **Methods, 2^nd^, 3^rd^ and 4^th^ paragraphs and supplementary file** |
| Bias | 9 | Describe any efforts to address potential sources of bias ‘**Sensitivity analysis’ paragraphs of Methods and Results** |
| Study size | 10 | Explain how the study size was arrived at **Methods, second paragraph** |
| Quantitative variables | 11 | Explain how quantitative variables were handled in the analyses. If applicable, describe which groupings were chosen and why **Methods 5^th^ paragraph** |
| Statistical methods | 12 | (*a*) Describe all statistical methods, including those used to control for confounding **Methods paragraph on ‘decision tree’** |
|  |  | (*b*) Describe any methods used to examine subgroups and interactions  **Methods paragraph on ‘decision tree’** |
|  |  | (*c*) Explain how missing data were addressed **Methods paragraph on ‘sensitivity analysis’** |
|  |  | (*d*) If applicable, explain how loss to follow-up was addressed **Not applicable** |
|  |  | (*e*) Describe any sensitivity analyses **Sensitivity analysis’ paragraphs of Methods and Results** |
| Results | | |
| Participants | 13* | (a) Report numbers of individuals at each stage of study—eg numbers potentially eligible, examined for eligibility, confirmed eligible, included in the study, completing follow-up, and analysed **Results paragraph one and Supplementary Figure 1** |
|  |  | (b) Give reasons for non-participation at each stage **Results paragraph one and Supplementary Figure 1** |
|  |  | (c) Consider use of a flow diagram **Supplementary Figure 1** |
| Descriptive data | 14* | (a) Give characteristics of study participants (eg demographic, clinical, social) and information on exposures and potential confounders **Table 2** |
|  |  | (b) Indicate number of participants with missing data for each variable of interest **Table 2** |
|  |  | (c) Summarise follow-up time (eg, average and total amount) **Supplementary Table 2.** |
| Outcome data | 15* | Report numbers of outcome events or summary measures over time **Table 2.** |
| Main results | 16 | (*a*) Give unadjusted estimates and, if applicable, confounder-adjusted estimates and their precision (eg, 95% confidence interval). Make clear which confounders were adjusted for and why they were included **Table 3, Figures 2-4** |
|  |  | (*b*) Report category boundaries when continuous variables were categorized **Table 3, Figures 2-4** |
|  |  | (*c*) If relevant, consider translating estimates of relative risk into absolute risk for a meaningful time period **Table 3, Figures 2-4** |
| Other analyses | 17 | Report other analyses done—eg analyses of subgroups and interactions, and sensitivity analyses **Results, ‘sensitivity analyses’** |
| Discussion | | |
| Key results | 18 | Summarise key results with reference to study objectives **Discussion paragraph one.** |
| Limitations | 19 | Discuss limitations of the study, taking into account sources of potential bias or imprecision. Discuss both direction and magnitude of any potential bias **Discussion, strengths and limitations section** |
| Interpretation | 20 | Give a cautious overall interpretation of results considering objectives, limitations, multiplicity of analyses, results from similar studies, and other relevant evidence **Discussion, conclusions section** |
| Generalisability | 21 | Discuss the generalisability (external validity) of the study results **Discussion, strengths and limitations section** |
| Other information | | |
| Funding | 22 | Give the source of funding and the role of the funders for the present study and, if applicable, for the original study on which the present article is based **Funding statement** |

*Give information separately for exposed and unexposed groups.

**Note:** An Explanation and Elaboration article discusses each checklist item and gives methodological background and published examples of transparent reporting. The STROBE checklist is best used in conjunction with this article (freely available on the Web sites of PLoS Medicine at http://www.plosmedicine.org/, Annals of Internal Medicine at http://www.annals.org/, and Epidemiology at http://www.epidem.com/). Information on the STROBE Initiative is available at http://www.strobe-statement.org.
