# Supplementary material for: Probability of sepsis after infection consultations in primary care in the United Kingdom in 2002–2017: Population-based cohort study and decision analytic model
Source: PLoS Med. 2020 Jul 23;17(7):e1003202. doi: 10.1371/journal.pmed.1003202 (PMC7377386; doi:10.1371/journal.pmed.1003202)
Supplement: S4 Table — (DOCX) [file pmed.1003202.s005.docx]

**S4 Table: Proportion of consultations with antibiotics prescribed and consultation rates per person year for different common infections.**

|  | | **Proportion of consultations with AB prescribed** | | | |  | **Consultation rate per patient year** | | | |  |
| --- | --- | --- | --- | --- | --- | --- | --- | --- | --- | --- | --- |
|  |  | **All** | **RTI** | **Skin** | **UTI** |  | **All** | **RTI** | **Skin** | **UTI** |  |
|  |  |  |  |  |  |  |  |  |  |  |  |
| **Male** | 0-4 | 0.43 | 0.43 | 0.54 | 0.55 |  | 1.310 | 1.233 | 0.069 | 0.011 |  |
|  | 5-14 | 0.48 | 0.47 | 0.63 | 0.68 |  | 0.428 | 0.381 | 0.044 | 0.005 |  |
|  | 15-24 | 0.58 | 0.56 | 0.68 | 0.74 |  | 0.232 | 0.196 | 0.034 | 0.003 |  |
|  | 25-34 | 0.60 | 0.58 | 0.69 | 0.73 |  | 0.186 | 0.152 | 0.032 | 0.003 |  |
|  | 35-44 | 0.62 | 0.59 | 0.71 | 0.78 |  | 0.201 | 0.163 | 0.032 | 0.006 |  |
|  | 45-54 | 0.62 | 0.59 | 0.72 | 0.77 |  | 0.216 | 0.175 | 0.033 | 0.008 |  |
|  | 55-64 | 0.63 | 0.61 | 0.71 | 0.78 |  | 0.284 | 0.233 | 0.037 | 0.014 |  |
|  | 65-74 | 0.64 | 0.61 | 0.71 | 0.79 |  | 0.391 | 0.319 | 0.045 | 0.028 |  |
|  | 75-84 | 0.63 | 0.60 | 0.71 | 0.77 |  | 0.473 | 0.363 | 0.060 | 0.052 |  |
|  | 85+ | 0.61 | 0.57 | 0.71 | 0.72 |  | 0.573 | 0.398 | 0.083 | 0.094 |  |
|  |  |  |  |  |  |  |  |  |  |  |  |
| **Female** | 0-4 | 0.43 | 0.42 | 0.52 | 0.64 |  | 1.215 | 1.120 | 0.062 | 0.035 |  |
|  | 5-14 | 0.51 | 0.48 | 0.62 | 0.76 |  | 0.494 | 0.422 | 0.045 | 0.029 |  |
|  | 15-24 | 0.61 | 0.55 | 0.66 | 0.86 |  | 0.481 | 0.364 | 0.045 | 0.073 |  |
|  | 25-34 | 0.63 | 0.58 | 0.68 | 0.87 |  | 0.458 | 0.343 | 0.047 | 0.070 |  |
|  | 35-44 | 0.66 | 0.61 | 0.72 | 0.88 |  | 0.434 | 0.327 | 0.047 | 0.062 |  |
|  | 45-54 | 0.66 | 0.61 | 0.73 | 0.88 |  | 0.424 | 0.321 | 0.044 | 0.060 |  |
|  | 55-64 | 0.67 | 0.62 | 0.72 | 0.89 |  | 0.479 | 0.363 | 0.046 | 0.072 |  |
|  | 65-74 | 0.67 | 0.61 | 0.72 | 0.88 |  | 0.539 | 0.391 | 0.052 | 0.099 |  |
|  | 75-84 | 0.66 | 0.59 | 0.73 | 0.85 |  | 0.564 | 0.371 | 0.067 | 0.128 |  |
|  | 85+ | 0.64 | 0.55 | 0.72 | 0.79 |  | 0.598 | 0.345 | 0.094 | 0.162 |  |
|  |  |  |  |  |  |  |  |  |  |  |  |
