# Supplementary material for: Probability of sepsis after infection consultations in primary care in the United Kingdom in 2002–2017: Population-based cohort study and decision analytic model
Source: PLoS Med. 2020 Jul 23;17(7):e1003202. doi: 10.1371/journal.pmed.1003202 (PMC7377386; doi:10.1371/journal.pmed.1003202)
Supplement: S5 Table — CPRD, Clinical Practice Research Datalink; PY, sum of person-years from 2002 to 2017. (DOCX) [file pmed.1003202.s006.docx]

**S5 Table: Estimated distribution of CPRD GOLD population by frailty level. PY, sum of person-years from 2002 to 2017.**

|  | **Age -group (years)** | **Frailty level** | | | | | | | | |
| --- | --- | --- | --- | --- | --- | --- | --- | --- | --- | --- |
| **Gender** |  | **Total** | **Fit** | | **Mild** | | **Moderate** | | **Severe** | |
|  |  | **PY** | **PY** | **%** | **PY** | **%** | **PY** | **%** | **PY** | **%** |
|  |  |  |  |  |  |  |  |  |  |  |
| Male | 55-64 | 4248353 | 3428566 | 80.7 | 708434.3 | 16.7 | 98301.92 | 2.3 | 13050.77 | 0.3 |
|  | 65-74 | 3078449 | 1872763 | 60.8 | 938048.1 | 30.5 | 224575.2 | 7.3 | 43063.57 | 1.4 |
|  | 75-84 | 1784234 | 685799.8 | 38.4 | 699969.2 | 39.2 | 306134.1 | 17.2 | 92331.3 | 5.2 |
|  | 85+ | 541225.5 | 131139.9 | 24.2 | 195808.9 | 36.2 | 145432.8 | 26.9 | 68843.92 | 12.7 |
|  |  |  |  |  |  |  |  |  |  |  |
| Female | 55-64 | 4218980 | 3022092 | 71.6 | 973288.8 | 23.1 | 193037.1 | 4.6 | 30561.39 | 0.7 |
|  | 65-74 | 3309699 | 1791749 | 54.1 | 1096905 | 33.1 | 340026.6 | 10.3 | 81018.27 | 2.4 |
|  | 75-84 | 2364536 | 800222.4 | 33.8 | 899877.3 | 38.1 | 480864.1 | 20.3 | 183572.5 | 7.8 |
|  | 85+ | 1141183 | 221669.4 | 19.4 | 389870.5 | 34.2 | 327228 | 28.7 | 202415.1 | 17.7 |
|  |  |  |  |  |  |  |  |  |  |  |
