# Supplementary material for: Probability of sepsis after infection consultations in primary care in the United Kingdom in 2002–2017: Population-based cohort study and decision analytic model
Source: PLoS Med. 2020 Jul 23;17(7):e1003202. doi: 10.1371/journal.pmed.1003202 (PMC7377386; doi:10.1371/journal.pmed.1003202)
Supplement: S6 Table — (DOCX) [file pmed.1003202.s007.docx]

**S6 Table: Distribution of sepsis cases by gender, region and period.**

|  |  | **Male** | **Female** |
| --- | --- | --- | --- |
|  |  |  |  |
| **Region** | North East | 375 | 361 |
|  | North West | 1,750 | 1,843 |
|  | Yorkshire & The Humber | 420 | 431 |
|  | East Midlands | 447 | 472 |
|  | West Midlands | 1,652 | 1,779 |
|  | East of England | 1,195 | 1,272 |
|  | South West | 1,280 | 1,235 |
|  | South Central | 1,813 | 1,810 |
|  | London | 1,236 | 1,384 |
|  | South East Coast | 1,688 | 1,777 |
|  | Northern Ireland | 899 | 935 |
|  | Scotland | 2,558 | 2,578 |
|  | Wales | 2,045 | 2,009 |
|  |  |  |  |
| **Period** | 2002-2005 | 2,703 | 3,090 |
|  | 2006-2009 | 3,780 | 4,043 |
|  | 2010-2013 | 5,093 | 5,007 |
|  | 2014-2017 | 5,782 | 5,746 |
|  |  |  |  |
