# Supplementary material for: Probability of sepsis after infection consultations in primary care in the United Kingdom in 2002–2017: Population-based cohort study and decision analytic model
Source: PLoS Med. 2020 Jul 23;17(7):e1003202. doi: 10.1371/journal.pmed.1003202 (PMC7377386; doi:10.1371/journal.pmed.1003202)
Supplement: S7 Table — (DOCX) [file pmed.1003202.s008.docx]

**S7 Table: Estimates by frailty category.**

| Gender | Age | Frailty | P(Infection) | P(Infection  \|Sepsis) | P(AB  \|Infection) | P(Sepsis\|[AB\| infection]) | | | P(Sepsis\|[NoAB\| infection]) | | | Number needed to treat (NNT) | | |
| --- | --- | --- | --- | --- | --- | --- | --- | --- | --- | --- | --- | --- | --- | --- |
|  |  |  |  |  |  | LL | Estimate | UL | LL | Estimate | UL | LL | Estimate | UL |
| Male | 55 | Non-frail | 0.016556 | 0.158349 | 0.623487 | 0.000056 | 0.000080 | 0.000109 | 0.000433 | 0.000514 | 0.000606 | 1890 | 2309 | 2879 |
| Male | 55 | Mild | 0.047358 | 0.199324 | 0.637353 | 0.000120 | 0.000163 | 0.000217 | 0.000845 | 0.000999 | 0.001169 | 989 | 1199 | 1490 |
| Male | 55 | Moderate | 0.082111 | 0.254672 | 0.623857 | 0.000210 | 0.000331 | 0.000494 | 0.001383 | 0.001779 | 0.002261 | 514 | 693 | 983 |
| Male | 55 | Severe | 0.081950 | 0.267330 | 0.654581 | 0.000312 | 0.000754 | 0.001484 | 0.003107 | 0.004823 | 0.007142 | 156 | 247 | 459 |
| Male | 65 | Non-frail | 0.018944 | 0.145885 | 0.629726 | 0.000074 | 0.000108 | 0.000152 | 0.000582 | 0.000705 | 0.000842 | 1354 | 1680 | 2133 |
| Male | 65 | Mild | 0.046372 | 0.175976 | 0.638661 | 0.000124 | 0.000164 | 0.000211 | 0.001029 | 0.001179 | 0.001343 | 845 | 986 | 1167 |
| Male | 65 | Moderate | 0.076233 | 0.210167 | 0.651978 | 0.000258 | 0.000349 | 0.000456 | 0.001860 | 0.002189 | 0.002540 | 454 | 544 | 669 |
| Male | 65 | Severe | 0.082096 | 0.253235 | 0.653231 | 0.000566 | 0.000866 | 0.001249 | 0.003722 | 0.004731 | 0.005947 | 196 | 259 | 360 |
| Male | 75 | Non-frail | 0.018461 | 0.179665 | 0.649718 | 0.000120 | 0.000194 | 0.000294 | 0.001093 | 0.001381 | 0.001727 | 650 | 843 | 1126 |
| Male | 75 | Mild | 0.040872 | 0.203532 | 0.634772 | 0.000234 | 0.000300 | 0.000379 | 0.001897 | 0.002138 | 0.002397 | 475 | 545 | 632 |
| Male | 75 | Moderate | 0.067689 | 0.235030 | 0.632469 | 0.000462 | 0.000573 | 0.000701 | 0.002695 | 0.003038 | 0.003414 | 350 | 406 | 477 |
| Male | 75 | Severe | 0.082126 | 0.291013 | 0.616987 | 0.001134 | 0.001408 | 0.001740 | 0.005003 | 0.005754 | 0.006606 | 191 | 230 | 283 |
| Male | 85 | Non-frail | 0.016099 | 0.197138 | 0.631600 | 0.000134 | 0.000337 | 0.000690 | 0.001884 | 0.002813 | 0.003972 | 274 | 407 | 677 |
| Male | 85 | Mild | 0.038496 | 0.254710 | 0.624595 | 0.000434 | 0.000620 | 0.000843 | 0.003845 | 0.004516 | 0.005277 | 214 | 257 | 313 |
| Male | 85 | Moderate | 0.063580 | 0.241235 | 0.613200 | 0.000634 | 0.000831 | 0.001064 | 0.003484 | 0.004060 | 0.004694 | 257 | 310 | 384 |
| Male | 85 | Severe | 0.082131 | 0.301107 | 0.600634 | 0.001144 | 0.001487 | 0.001890 | 0.006139 | 0.007076 | 0.008121 | 149 | 179 | 218 |
| Female | 55 | Non-frail | 0.025727 | 0.138552 | 0.668989 | 0.000038 | 0.000054 | 0.000075 | 0.000260 | 0.000319 | 0.000386 | 3001 | 3782 | 4907 |
| Female | 55 | Mild | 0.064976 | 0.184847 | 0.670187 | 0.000062 | 0.000084 | 0.000111 | 0.000428 | 0.000511 | 0.000605 | 1909 | 2347 | 2951 |
| Female | 55 | Moderate | 0.082123 | 0.257667 | 0.674641 | 0.000171 | 0.000247 | 0.000342 | 0.000801 | 0.001030 | 0.001304 | 938 | 1281 | 1883 |
| Female | 55 | Severe | 0.082055 | 0.264378 | 0.704832 | 0.000251 | 0.000477 | 0.000833 | 0.002323 | 0.003413 | 0.004735 | 234 | 343 | 556 |
| Female | 65 | Non-frail | 0.024251 | 0.145675 | 0.675931 | 0.000054 | 0.000079 | 0.000111 | 0.000356 | 0.000447 | 0.000556 | 2089 | 2718 | 3697 |
| Female | 65 | Mild | 0.056554 | 0.173590 | 0.665533 | 0.000086 | 0.000112 | 0.000145 | 0.000543 | 0.000637 | 0.000741 | 1583 | 1909 | 2347 |
| Female | 65 | Moderate | 0.082130 | 0.253030 | 0.671455 | 0.000185 | 0.000243 | 0.000311 | 0.001103 | 0.001306 | 0.001526 | 775 | 943 | 1180 |
| Female | 65 | Severe | 0.082112 | 0.278281 | 0.668612 | 0.000556 | 0.000763 | 0.001013 | 0.002424 | 0.003050 | 0.003765 | 329 | 438 | 624 |
| Female | 75 | Non-frail | 0.019766 | 0.178667 | 0.666323 | 0.000048 | 0.000089 | 0.000150 | 0.000594 | 0.000789 | 0.001029 | 1063 | 1432 | 2020 |
| Female | 75 | Mild | 0.043909 | 0.203145 | 0.664169 | 0.000121 | 0.000161 | 0.000209 | 0.001042 | 0.001198 | 0.001377 | 820 | 965 | 1150 |
| Female | 75 | Moderate | 0.073872 | 0.223524 | 0.663634 | 0.000198 | 0.000252 | 0.000314 | 0.001338 | 0.001530 | 0.001742 | 669 | 783 | 930 |
| Female | 75 | Severe | 0.082129 | 0.260897 | 0.657275 | 0.000457 | 0.000583 | 0.000732 | 0.003010 | 0.003460 | 0.003935 | 296 | 348 | 416 |
| Female | 85 | Non-frail | 0.015499 | 0.220584 | 0.648021 | 0.000123 | 0.000278 | 0.000523 | 0.001601 | 0.002287 | 0.003162 | 346 | 499 | 780 |
| Female | 85 | Mild | 0.035575 | 0.203488 | 0.644060 | 0.000261 | 0.000362 | 0.000489 | 0.002068 | 0.002442 | 0.002852 | 400 | 481 | 593 |
| Female | 85 | Moderate | 0.059508 | 0.271667 | 0.637731 | 0.000427 | 0.000538 | 0.000662 | 0.002839 | 0.003195 | 0.003590 | 326 | 376 | 439 |
| Female | 85 | Severe | 0.082132 | 0.295781 | 0.640735 | 0.000597 | 0.000735 | 0.000896 | 0.003748 | 0.004206 | 0.004703 | 251 | 288 | 335 |
