# Supplementary material for: Probability of sepsis after infection consultations in primary care in the United Kingdom in 2002–2017: Population-based cohort study and decision analytic model
Source: PLoS Med. 2020 Jul 23;17(7):e1003202. doi: 10.1371/journal.pmed.1003202 (PMC7377386; doi:10.1371/journal.pmed.1003202)
Supplement: S8 Table — (DOCX) [file pmed.1003202.s009.docx]

**S8 Table: Estimates by type of infection consultation.**

| Type | Gender | Age | p(Infection) | P(Infection\|  Sepsis) | P(AB\|Infection) | P(Sepsis\|[AB\| infection]) | | | P(Sepsis\|[NoAB\| infection]) | | | Number needed to treat (NNT) | | |
| --- | --- | --- | --- | --- | --- | --- | --- | --- | --- | --- | --- | --- | --- | --- |
|  |  |  |  |  |  | LL | Estimate | UL | LL | Estimate | UL | LL | Estimate | UL |
| RTI | Male | 0 | 0.082135 | 0.206974 | 0.428153 | 0.000009 | 0.000019 | 0.000032 | 0.000034 | 0.000048 | 0.000065 | 20396 | 34204 | 93441 |
| RTI | Male | 5 | 0.031328 | 0.143416 | 0.466181 | 0.000002 | 0.000006 | 0.000014 | 0.000034 | 0.000048 | 0.000064 | 17212 | 24471 | 38725 |
| RTI | Male | 15 | 0.016127 | 0.103858 | 0.556818 | 0.000016 | 0.000029 | 0.000049 | 0.000049 | 0.000074 | 0.000107 | 12438 | 22415 | 70856 |
| RTI | Male | 25 | 0.012450 | 0.110281 | 0.580357 | 0.000013 | 0.000026 | 0.000046 | 0.000109 | 0.000150 | 0.000202 | 5614 | 8071 | 12750 |
| RTI | Male | 35 | 0.013414 | 0.086743 | 0.591469 | 0.000011 | 0.000022 | 0.000037 | 0.000131 | 0.000172 | 0.000220 | 5015 | 6683 | 9413 |
| RTI | Male | 45 | 0.014394 | 0.099568 | 0.591907 | 0.000022 | 0.000036 | 0.000055 | 0.000267 | 0.000323 | 0.000385 | 2848 | 3493 | 4388 |
| RTI | Male | 55 | 0.019170 | 0.103201 | 0.607528 | 0.000054 | 0.000074 | 0.000098 | 0.000468 | 0.000539 | 0.000616 | 1840 | 2152 | 2565 |
| RTI | Male | 65 | 0.026178 | 0.102125 | 0.614425 | 0.000078 | 0.000102 | 0.000130 | 0.000803 | 0.000898 | 0.000998 | 1112 | 1257 | 1434 |
| RTI | Male | 75 | 0.029798 | 0.122233 | 0.604233 | 0.000219 | 0.000267 | 0.000322 | 0.001691 | 0.001857 | 0.002024 | 567 | 629 | 706 |
| RTI | Male | 85 | 0.032713 | 0.131860 | 0.570078 | 0.000330 | 0.000437 | 0.000564 | 0.002955 | 0.003316 | 0.003711 | 304 | 348 | 400 |
| RTI | Female | 0 | 0.082135 | 0.212896 | 0.418881 | 0.000008 | 0.000016 | 0.000029 | 0.000034 | 0.000048 | 0.000067 | 19141 | 31601 | 78847 |
| RTI | Female | 5 | 0.034661 | 0.102811 | 0.481292 | 0.000004 | 0.000010 | 0.000018 | 0.000012 | 0.000021 | 0.000032 | NA | 87821 | 578341 |
| RTI | Female | 15 | 0.029911 | 0.078999 | 0.550079 | 0.000008 | 0.000015 | 0.000026 | 0.000035 | 0.000051 | 0.000072 | 17347 | 27818 | 59722 |
| RTI | Female | 25 | 0.028166 | 0.075068 | 0.576954 | 0.000010 | 0.000018 | 0.000029 | 0.000058 | 0.000078 | 0.000102 | 11668 | 16794 | 26976 |
| RTI | Female | 35 | 0.026890 | 0.085332 | 0.607250 | 0.000010 | 0.000017 | 0.000026 | 0.000096 | 0.000122 | 0.000151 | 7396 | 9573 | 12867 |
| RTI | Female | 45 | 0.026393 | 0.087431 | 0.605222 | 0.000023 | 0.000034 | 0.000047 | 0.000150 | 0.000182 | 0.000217 | 5401 | 6760 | 8771 |
| RTI | Female | 55 | 0.029803 | 0.113548 | 0.622262 | 0.000051 | 0.000066 | 0.000083 | 0.000324 | 0.000371 | 0.000425 | 2778 | 3271 | 3910 |
| RTI | Female | 65 | 0.032074 | 0.105860 | 0.612023 | 0.000074 | 0.000093 | 0.000115 | 0.000471 | 0.000532 | 0.000600 | 1966 | 2278 | 2686 |
| RTI | Female | 75 | 0.030502 | 0.111674 | 0.588035 | 0.000143 | 0.000177 | 0.000216 | 0.000875 | 0.000974 | 0.001081 | 1103 | 1255 | 1446 |
| RTI | Female | 85 | 0.028315 | 0.115654 | 0.549517 | 0.000253 | 0.000322 | 0.000404 | 0.001858 | 0.002068 | 0.002287 | 506 | 573 | 658 |
| Skin | Male | 0 | 0.005665 | 0.011818 | 0.542488 | 0.000003 | 0.000025 | 0.000090 | 0.000004 | 0.000029 | 0.000109 | NA | 18132 | 707630 |
| Skin | Male | 5 | 0.003573 | 0.018749 | 0.626287 | 0.000002 | 0.000013 | 0.000045 | 0.000020 | 0.000061 | 0.000141 | NA | 20815 | 166278 |
| Skin | Male | 15 | 0.002792 | 0.051507 | 0.682852 | 0.000043 | 0.000092 | 0.000168 | 0.000123 | 0.000242 | 0.000419 | 2590 | 6592 | 42784 |
| Skin | Male | 25 | 0.002617 | 0.050392 | 0.691383 | 0.000029 | 0.000070 | 0.000135 | 0.000231 | 0.000389 | 0.000616 | 1823 | 3165 | 6827 |
| Skin | Male | 35 | 0.002655 | 0.037531 | 0.713387 | 0.000019 | 0.000047 | 0.000098 | 0.000333 | 0.000510 | 0.000744 | 1437 | 2176 | 3578 |
| Skin | Male | 45 | 0.002714 | 0.048882 | 0.719372 | 0.000069 | 0.000123 | 0.000196 | 0.000826 | 0.001101 | 0.001434 | 760 | 1025 | 1444 |
| Skin | Male | 55 | 0.003078 | 0.041221 | 0.708535 | 0.000108 | 0.000174 | 0.000262 | 0.001401 | 0.001763 | 0.002180 | 497 | 630 | 821 |
| Skin | Male | 65 | 0.003729 | 0.029168 | 0.712988 | 0.000161 | 0.000247 | 0.000356 | 0.001810 | 0.002239 | 0.002741 | 398 | 503 | 646 |
| Skin | Male | 75 | 0.004905 | 0.028350 | 0.706398 | 0.000419 | 0.000575 | 0.000766 | 0.002352 | 0.002911 | 0.003570 | 332 | 430 | 572 |
| Skin | Male | 85 | 0.006786 | 0.025088 | 0.711236 | 0.000303 | 0.000515 | 0.000805 | 0.003034 | 0.004018 | 0.005210 | 212 | 286 | 402 |
| Skin | Female | 0 | 0.005114 | 0.027659 | 0.520371 | 0.000013 | 0.000056 | 0.000157 | 0.000023 | 0.000085 | 0.000211 | NA | 12905 | 295022 |
| Skin | Female | 5 | 0.003668 | 0.027834 | 0.617105 | 0.000002 | 0.000014 | 0.000048 | 0.000028 | 0.000077 | 0.000165 | 5849 | 16033 | 94672 |
| Skin | Female | 15 | 0.003690 | 0.031126 | 0.660092 | 0.000020 | 0.000051 | 0.000106 | 0.000095 | 0.000190 | 0.000329 | 3493 | 7347 | 29880 |
| Skin | Female | 25 | 0.003896 | 0.028048 | 0.683214 | 0.000025 | 0.000056 | 0.000105 | 0.000140 | 0.000245 | 0.000391 | 2966 | 5296 | 13657 |
| Skin | Female | 35 | 0.003824 | 0.036134 | 0.716201 | 0.000033 | 0.000064 | 0.000111 | 0.000304 | 0.000447 | 0.000627 | 1772 | 2620 | 4274 |
| Skin | Female | 45 | 0.003638 | 0.029238 | 0.726441 | 0.000039 | 0.000074 | 0.000124 | 0.000439 | 0.000615 | 0.000847 | 1292 | 1852 | 2810 |
| Skin | Female | 55 | 0.003754 | 0.025652 | 0.724041 | 0.000081 | 0.000132 | 0.000199 | 0.000603 | 0.000826 | 0.001093 | 1034 | 1447 | 2149 |
| Skin | Female | 65 | 0.004243 | 0.030651 | 0.723396 | 0.000148 | 0.000222 | 0.000314 | 0.001186 | 0.001498 | 0.001878 | 602 | 784 | 1051 |
| Skin | Female | 75 | 0.005536 | 0.032319 | 0.725883 | 0.000153 | 0.000229 | 0.000327 | 0.001914 | 0.002333 | 0.002814 | 386 | 476 | 598 |
| Skin | Female | 85 | 0.007704 | 0.041203 | 0.719492 | 0.000399 | 0.000549 | 0.000732 | 0.003107 | 0.003758 | 0.004504 | 252 | 312 | 394 |
| UTI | Male | 0 | 0.000873 | 0.020844 | 0.553835 | 0.000002 | 0.000074 | 0.000411 | 0.000198 | 0.000627 | 0.001469 | 618 | 1883 | 11322 |
| UTI | Male | 5 | 0.000373 | 0.008833 | 0.682663 | 0.000001 | 0.000041 | 0.000229 | 0.000065 | 0.000336 | 0.001024 | NA | 3371 | 33720 |
| UTI | Male | 15 | 0.000220 | 0.012931 | 0.743315 | 0.000026 | 0.000179 | 0.000626 | 0.000314 | 0.001145 | 0.002897 | 308 | 1050 | 7539 |
| UTI | Male | 25 | 0.000282 | 0.021392 | 0.732734 | 0.000095 | 0.000313 | 0.000768 | 0.000651 | 0.001581 | 0.003206 | 334 | 800 | 3591 |
| UTI | Male | 35 | 0.000494 | 0.027367 | 0.783120 | 0.000109 | 0.000267 | 0.000522 | 0.001293 | 0.002274 | 0.003662 | 295 | 501 | 1020 |
| UTI | Male | 45 | 0.000676 | 0.037812 | 0.771271 | 0.000285 | 0.000488 | 0.000769 | 0.002633 | 0.003747 | 0.005200 | 212 | 308 | 473 |
| UTI | Male | 55 | 0.001125 | 0.052882 | 0.776441 | 0.000589 | 0.000825 | 0.001119 | 0.005748 | 0.007133 | 0.008782 | 125 | 159 | 205 |
| UTI | Male | 65 | 0.002300 | 0.058626 | 0.790291 | 0.000721 | 0.000931 | 0.001180 | 0.007847 | 0.009227 | 0.010759 | 102 | 121 | 145 |
| UTI | Male | 75 | 0.004247 | 0.088352 | 0.768632 | 0.001090 | 0.001345 | 0.001634 | 0.013504 | 0.015183 | 0.016956 | 64 | 72 | 82 |
| UTI | Male | 85 | 0.007757 | 0.121007 | 0.717867 | 0.001795 | 0.002243 | 0.002755 | 0.015119 | 0.017252 | 0.019562 | 58 | 67 | 78 |
| UTI | Female | 0 | 0.002914 | 0.042357 | 0.637653 | 0.000020 | 0.000086 | 0.000234 | 0.000152 | 0.000373 | 0.000749 | 1325 | 3495 | 19928 |
| UTI | Female | 5 | 0.002366 | 0.015406 | 0.761274 | 0.000002 | 0.000016 | 0.000057 | 0.000017 | 0.000081 | 0.000239 | NA | 14683 | 161424 |
| UTI | Female | 15 | 0.006035 | 0.049219 | 0.862441 | 0.000024 | 0.000047 | 0.000081 | 0.000229 | 0.000404 | 0.000649 | 1662 | 2803 | 5611 |
| UTI | Female | 25 | 0.005716 | 0.040446 | 0.867110 | 0.000045 | 0.000073 | 0.000114 | 0.000218 | 0.000379 | 0.000608 | 1867 | 3302 | 7226 |
| UTI | Female | 35 | 0.005067 | 0.046118 | 0.880630 | 0.000037 | 0.000061 | 0.000096 | 0.000664 | 0.000941 | 0.001301 | 807 | 1139 | 1668 |
| UTI | Female | 45 | 0.004915 | 0.054310 | 0.884879 | 0.000081 | 0.000119 | 0.000166 | 0.001331 | 0.001752 | 0.002247 | 468 | 613 | 826 |
| UTI | Female | 55 | 0.005930 | 0.050594 | 0.887941 | 0.000086 | 0.000123 | 0.000170 | 0.002125 | 0.002635 | 0.003235 | 321 | 398 | 501 |
| UTI | Female | 65 | 0.008093 | 0.071785 | 0.876056 | 0.000215 | 0.000271 | 0.000335 | 0.003202 | 0.003787 | 0.004409 | 241 | 284 | 342 |
| UTI | Female | 75 | 0.010477 | 0.089833 | 0.849675 | 0.000237 | 0.000299 | 0.000368 | 0.005478 | 0.006197 | 0.006947 | 150 | 170 | 193 |
| UTI | Female | 85 | 0.013325 | 0.118305 | 0.788903 | 0.000521 | 0.000647 | 0.000789 | 0.008076 | 0.009001 | 0.010018 | 107 | 120 | 135 |
