# Supplementary material for: Probability of sepsis after infection consultations in primary care in the United Kingdom in 2002–2017: Population-based cohort study and decision analytic model
Source: PLoS Med. 2020 Jul 23;17(7):e1003202. doi: 10.1371/journal.pmed.1003202 (PMC7377386; doi:10.1371/journal.pmed.1003202)
Supplement: S9 Table — Column headings as main text Table 2. (DOCX) [file pmed.1003202.s010.docx]

**S9 Table: Sensitivity analysis using data for 2014 to 2017 only. Column headings as main text Table 2.**

| **Gender** | **Age-group** | **P(Infection)** | **P(Sepsis)** | **P(Infection \| Sepsis)** | **P(AB \| Infection)** | **P(Sepsis\|[No AB\|Infection]** | **P(Sepsis\|**  **[AB\|Infection]** | **NNT** | **LL 95%UI** | **UL 95% UI** |
| --- | --- | --- | --- | --- | --- | --- | --- | --- | --- | --- |
|  |  |  |  |  |  |  |  |  |  |  |
| **Male** | 0 | 0.08 | 0.000019 | 0.26 | 0.43 | 0.000091 | 0.000015 | 13522 | 7292 | 41501 |
|  | 5 | 0.03 | 0.000007 | 0.20 | 0.45 | 0.000078 | 0.000013 | 15757 | 8373 | 47247 |
|  | 15 | 0.02 | 0.000010 | 0.16 | 0.53 | 0.000103 | 0.000092 | 12068 | -318568 | 341555 |
|  | 25 | 0.01 | 0.000012 | 0.20 | 0.56 | 0.000357 | 0.000050 | 3299 | 2045 | 6375 |
|  | 35 | 0.01 | 0.000022 | 0.15 | 0.58 | 0.000387 | 0.000082 | 3317 | 2103 | 6525 |
|  | 45 | 0.02 | 0.000034 | 0.18 | 0.59 | 0.000763 | 0.000120 | 1562 | 1164 | 2198 |
|  | 55 | 0.02 | 0.000089 | 0.19 | 0.57 | 0.001345 | 0.000316 | 973 | 768 | 1284 |
|  | 65 | 0.03 | 0.000188 | 0.17 | 0.61 | 0.002159 | 0.000377 | 561 | 473 | 677 |
|  | 75 | 0.04 | 0.000411 | 0.20 | 0.61 | 0.004157 | 0.000795 | 298 | 256 | 352 |
|  | 85 | 0.05 | 0.000741 | 0.23 | 0.61 | 0.007287 | 0.001290 | 167 | 141 | 202 |
|  |  |  |  |  |  |  |  |  |  |  |
| **Female** | 0 | 0.08 | 0.000019 | 0.19 | 0.44 | 0.000048 | 0.000043 | 22082 | -562694 | 627933 |
|  | 5 | 0.03 | 0.000005 | 0.23 | 0.48 | 0.000049 | 0.000016 | 29688 | -141155 | 221992 |
|  | 15 | 0.04 | 0.000020 | 0.18 | 0.58 | 0.000169 | 0.000051 | 8603 | 4895 | 22552 |
|  | 25 | 0.03 | 0.000031 | 0.13 | 0.61 | 0.000212 | 0.000054 | 6401 | 4025 | 12955 |
|  | 35 | 0.03 | 0.000032 | 0.20 | 0.63 | 0.000387 | 0.000080 | 3277 | 2336 | 5025 |
|  | 45 | 0.03 | 0.000047 | 0.18 | 0.63 | 0.000517 | 0.000105 | 2427 | 1835 | 3407 |
|  | 55 | 0.04 | 0.000083 | 0.18 | 0.65 | 0.000770 | 0.000213 | 1795 | 1370 | 2534 |
|  | 65 | 0.04 | 0.000142 | 0.21 | 0.64 | 0.001322 | 0.000344 | 1024 | 828 | 1306 |
|  | 75 | 0.05 | 0.000258 | 0.22 | 0.65 | 0.002529 | 0.000391 | 468 | 400 | 552 |
|  | 85 | 0.05 | 0.000458 | 0.24 | 0.64 | 0.004775 | 0.000839 | 254 | 216 | 302 |
|  |  |  |  |  |  |  |  |  |  |  |
