# Supplementary material for: Probability of sepsis after infection consultations in primary care in the United Kingdom in 2002–2017: Population-based cohort study and decision analytic model
Source: PLoS Med. 2020 Jul 23;17(7):e1003202. doi: 10.1371/journal.pmed.1003202 (PMC7377386; doi:10.1371/journal.pmed.1003202)
Supplement: S1 Fig — (DOCX) [file pmed.1003202.s011.docx]

**S1 Fig: Flow chart showing participant selection for main and linked samples.**

**Hospital Episode Statistics (HES) Admitted Patient Care (APC)**

**ONS Mortality Statistics**

**CPRD GOLD 1987 to 2020:**

887 UK general practices

18,438,640 patients

35,244 first sepsis events in primary care records in 66.2 million patient-years

**CPRD GOLD 2002 to 2017:**

706 UK general practices

9,779,969 patients

Random sampling

Sample data for 671,830 patients used to estimate consultation and antibiotic prescribing rates

42,785 first sepsis events including 17,341 from primary care records, 17,363 from HES APC primary diagnoses and 8,081 in 36.2 million patient years follow-up

**CPRD GOLD Linkage Eligible 2002 to 2017:**

378 English general practices

5,524,983 patients
