# Supplementary material for: Probability of sepsis after infection consultations in primary care in the United Kingdom in 2002–2017: Population-based cohort study and decision analytic model
Source: PLoS Med. 2020 Jul 23;17(7):e1003202. doi: 10.1371/journal.pmed.1003202 (PMC7377386; doi:10.1371/journal.pmed.1003202)

**S2 Fig: Estimates for number of antibiotic prescriptions needed to prevent one sepsis episode (NNT) for four periods: 2002-2005 (blue), 2006 to 2009 (green), 2010 to 2013 (orange) and 2014 to 2017 (red).**


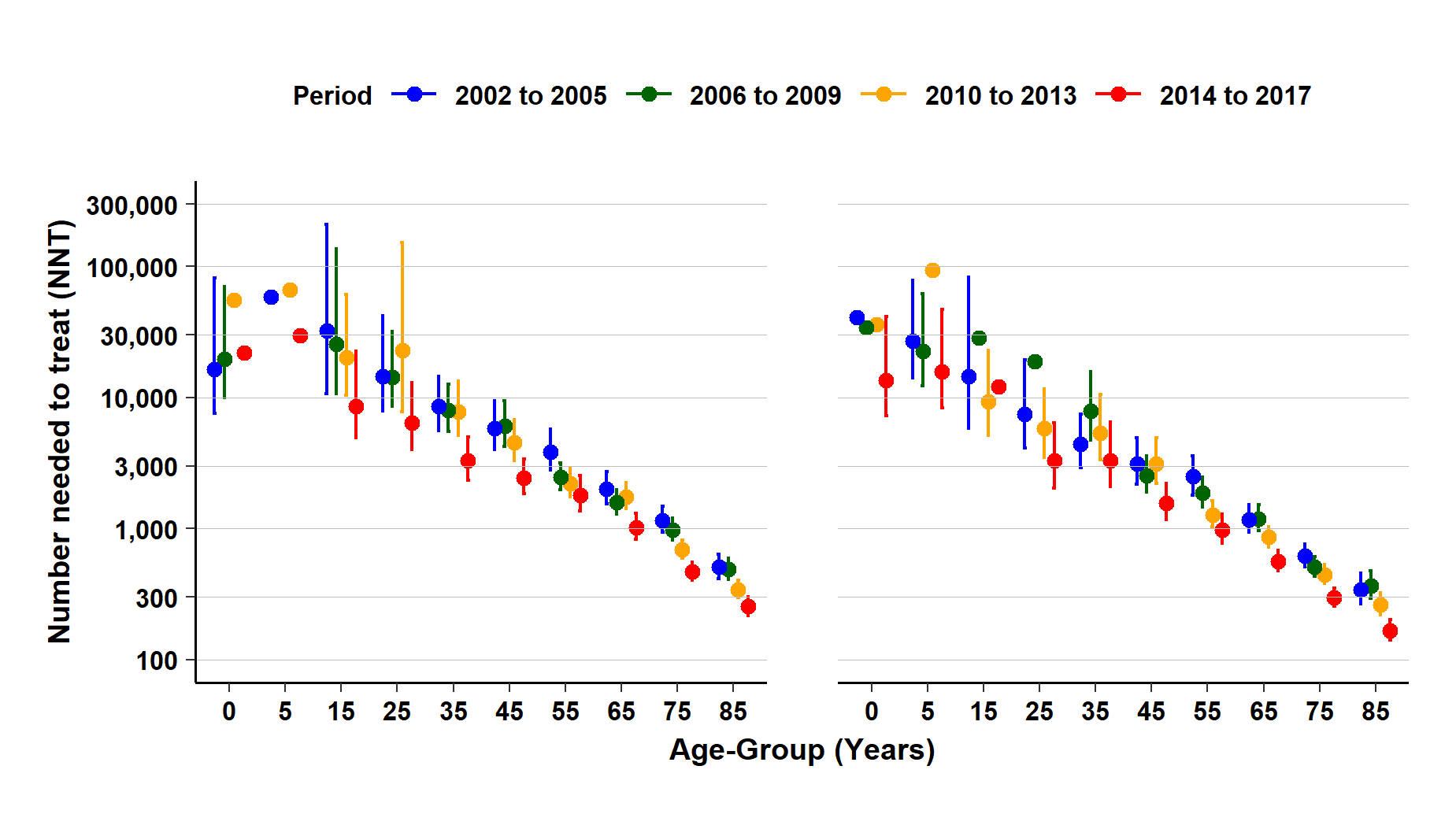

Supplement: S2 Fig — NNT, number needed to treat. (DOCX) [file pmed.1003202.s012.docx]
