# Supplementary material for: Probability of sepsis after infection consultations in primary care in the United Kingdom in 2002–2017: Population-based cohort study and decision analytic model
Source: PLoS Med. 2020 Jul 23;17(7):e1003202. doi: 10.1371/journal.pmed.1003202 (PMC7377386; doi:10.1371/journal.pmed.1003202)

**S3 Fig: Probability of an infection consultation in primary care in the 30 days preceding a sepsis diagnosis using CPRD (linked sample) records (red), CPRD and linked HES records (blue), or CPRD, HES and linked ONS mortality records (green).**


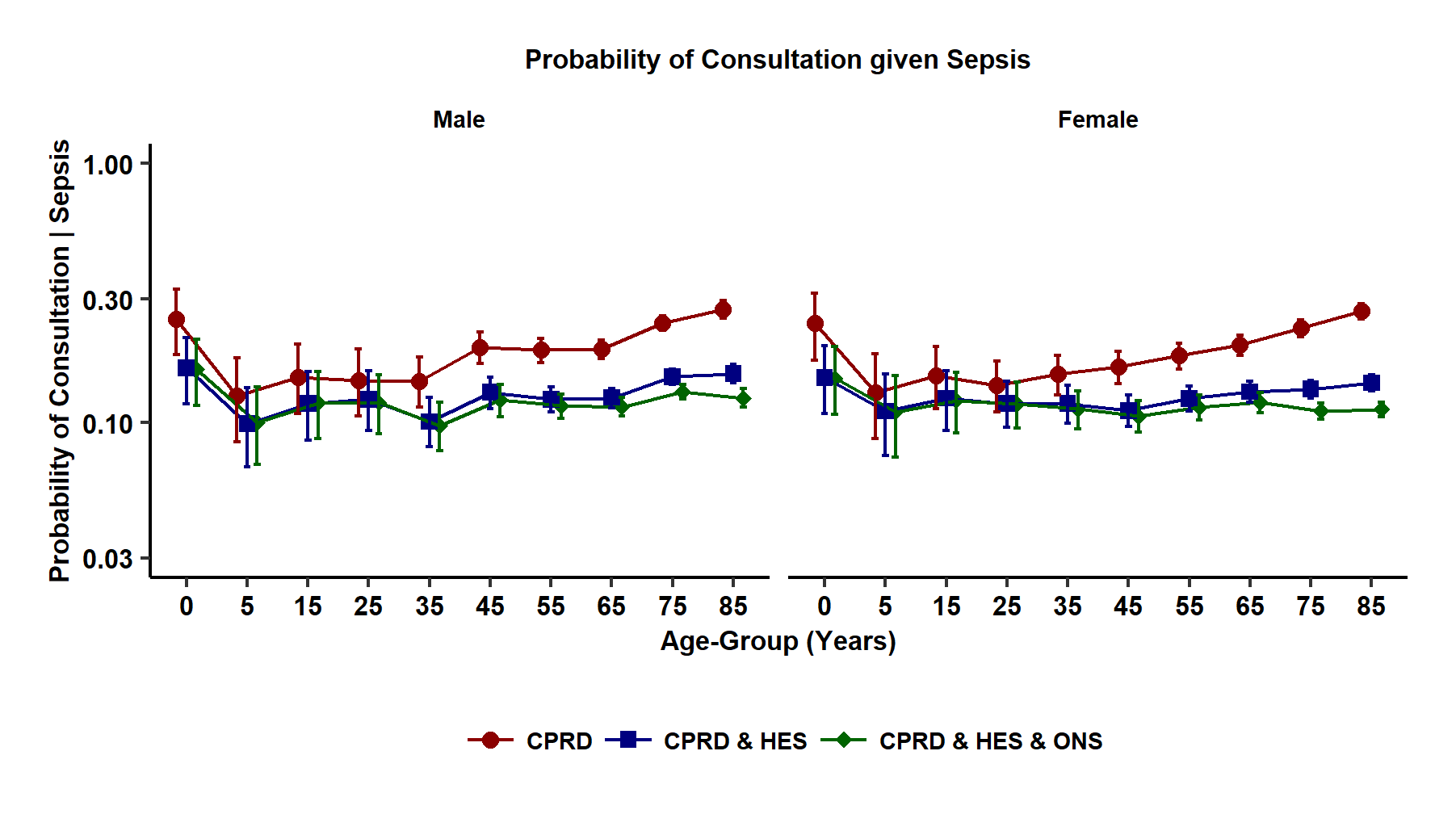

Supplement: S3 Fig — CPRD, Clinical Practice Research Datalink; HES, Hospital Episode Statistics; ONS, Office for National Statistics. (DOCX) [file pmed.1003202.s013.docx]
