# Supplementary material for: Probability of sepsis after infection consultations in primary care in the United Kingdom in 2002–2017: Population-based cohort study and decision analytic model
Source: PLoS Med. 2020 Jul 23;17(7):e1003202. doi: 10.1371/journal.pmed.1003202 (PMC7377386; doi:10.1371/journal.pmed.1003202)

**S4 Fig: Estimated probability (95% uncertainty interval) of a first sepsis event within 30 days of an infection consultation in primary care if antibiotics prescribed. CPRD (linked sample) records only (red), CPRD and linked HES records (blue), or CPRD, HES and linked ONS mortality records (green).**


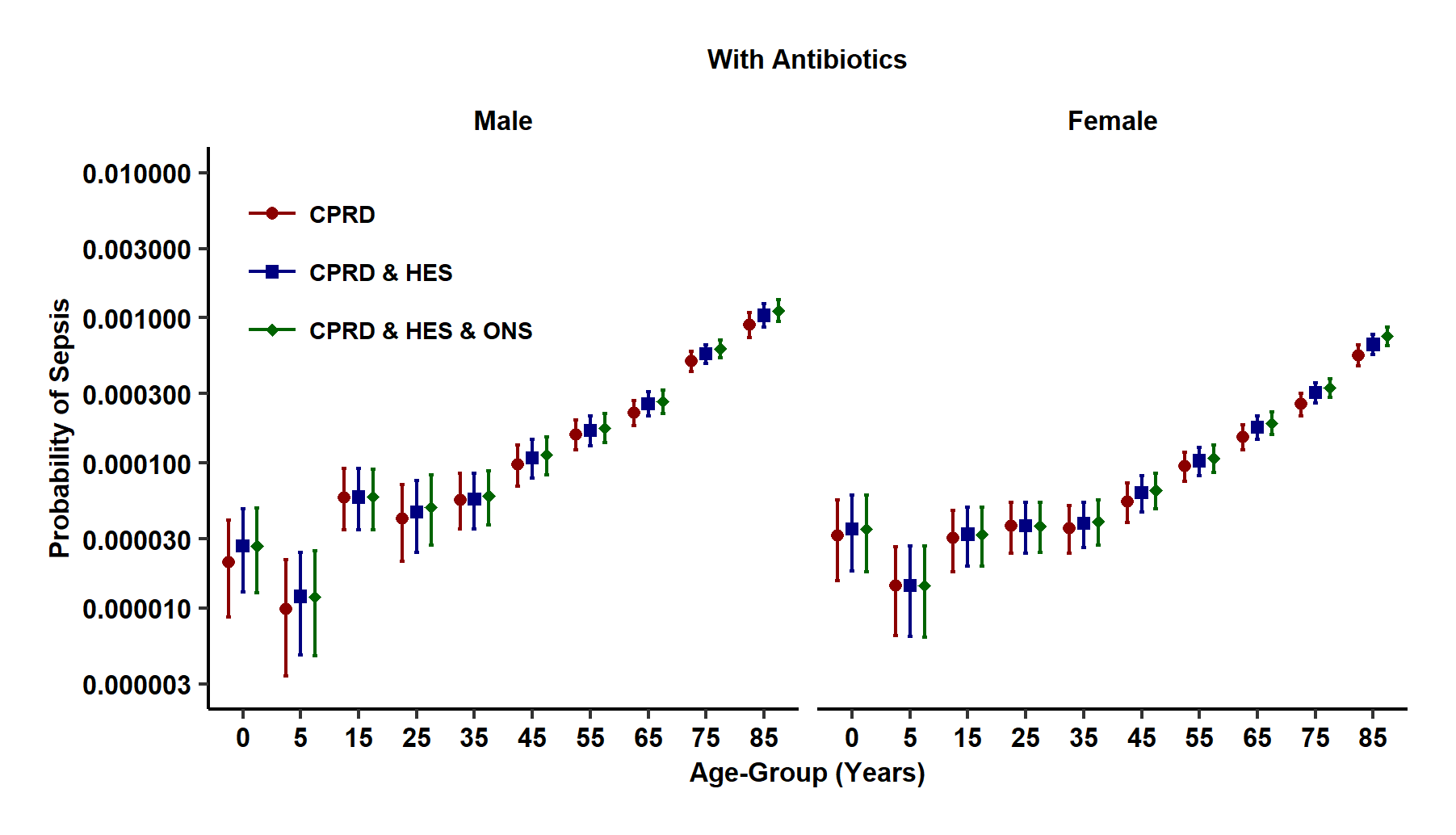

Supplement: S4 Fig — CPRD (linked sample) records only (red); CPRD and linked HES records (blue); or CPRD, HES, and linked ONS mortality records (green). CPRD, Clinical Practice Research Datalink; HES, Hospital Episode Statistics; ONS, Office for National Statistics. (DOCX) [file pmed.1003202.s014.docx]
