# Supplementary material for: Probability of sepsis after infection consultations in primary care in the United Kingdom in 2002–2017: Population-based cohort study and decision analytic model
Source: PLoS Med. 2020 Jul 23;17(7):e1003202. doi: 10.1371/journal.pmed.1003202 (PMC7377386; doi:10.1371/journal.pmed.1003202)
Supplement: Attachment — Submitted filename: ResponseToReviewers18May2020.docx [file pmed.1003202.s016.docx]

**Probability of sepsis after infection consultations in primary care in the UK. Population based cohort study and decision analytic model (PMEDICINE-D-20-01208R1)**

**Requests from the editors:**

*1. We ask that you address all points raised by the 3 reviewers, but want to draw particular attention to the following: Please address the reviewers’ requests regarding better reporting of your methodology.*

Thank you, we have provided additional methodological explanation and data, which we believe makes the analysis fully transparent.

*In particular, we ask that you provide a detailed response to the comments of reviewer #3 on the suitability of the dataset for your analyses.*

Thank you for this comment. For the primary analysis, we used a dataset for the 16-year period from 2002 to 2017 to provide a sufficiently large dataset to evaluate important sub-groups of age, frailty and infection type. The main concern of reviewer 3 focuses on changes over time in the definition and use of the term ‘sepsis’, as well as changes over time in antibiotic utilisation. To address this, we have repeated the primary analysis, using data only for the first four-year period (2002 to 2005) or the last four-year period (2014 to 2017) as sensitivity analyses (Supplementary Figure 2). We discuss these further in our response to reviewer 3.

*Please also consider the comments of reviewer #2 on whether it would be possible to include analyses by antibiotic class, though we recognise that this may not be feasible at this stage.*
Thank you, we agree with the reviewer that there have been changes in the use of broad-spectrum antibiotics, with prescribing generally becoming slightly more selective over time. Our approach does not readily lend itself to comparing different classes of antibiotics. We acknowledge this on page 14 (Discussion), where we now say: ’We also acknowledge that in addition to changes in overall antibiotic utilisation, there have been changes in the proportion of prescriptions for broad-spectrum antibiotics. Future studies might be designed to compare the probability if sepsis if broad-spectrum or narrow-spectrum antibiotics are prescribed.’

*2. Please revise your title according to PLOS Medicine's style, using sentence case and a colon before the study type. Please also mention that the study is based on data from the UK.*
Thank you, the title has been revised as requested.

*3. Please structure your abstract using the PLOS Medicine headings (Background, Methods and Findings, Conclusions). Please combine the Design, Setting, Participants etc. sections into one section: “Methods and findings”. In the last sentence of the Methods and Findings section, please describe the main limitation(s) of the study's methodology. Please also include summary demographic information (eg. age, sex) for the cohort as a whole.*
Thank you, the Abstract has been revised as requested. We now add ‘There were 35,244 first episodes of sepsis (17,886, 51%, female; median age 71 years, interquartile range 57 to 82 years).’

*4. Please use the term ‘patient’ rather than ‘participant’ throughout your manuscript.*
Thank you, this has been changed throughout.

*5. The Data Availability Statement (DAS) requires revision. For each data source used in your study: If the data are owned by a third party but available upon request, please note this and state the owner of the data set and contact information for data requests (web or email address). Note that a study author cannot be the contact person for the data. If the data are not freely available, please describe briefly the ethical, legal, or contractual restriction that prevents you from sharing it.*
Thank you, the Data Availability Statement has been revised as requested.

*6. At this stage, we ask that you include a short, non-technical Author Summary of your research to make findings accessible to a wide audience that includes both scientists and non-scientists.*

Thank you, an Author Summary has now been added.

*7. Please insert your citations before punctuation marks, eg: “…is attracting the concern of national governments and international organisations [1].” Please also ensure that your reference list is presented in the Vancouver style, including bold and italic formatting, where appropriate.*
Thank you, the reference list and citations have been revised as requested.

*8. Please include a full list of the read codes used in your study, as Supporting Information.*
Thank you, we now provide a full list of Read codes as a supplementary file.

*9. Please provide line numbers throughout your manuscript text.*
Thank you, line numbers have now been added.

*10. In the Abstract and Main Text, please include significance p values for the numerical data presented.*
Thank you, the analytical approach aimed to estimate probabilities, and associated 95% uncertainty intervals, using Bayes theorem. The method of null-hypothesis significance testing was not employed and there are no P values to report. We comment on this in the Discussion (p14), where we add: ‘We did not employ the approach of null-hypothesis significance-testing and do not report P values. Readers may wish to reflect on the substantive importance of estimated differences, and associated uncertainty intervals, for their work.’

*11. Please provide a table showing the baseline characteristics of the study population.*
Thank you, Table 2 has been transferred from the Supplementary file to the Main Text file. This shows the distribution of sepsis patients by age and gender.

*12. Please ensure that the study is reported according to the STROBE guidelines and include the completed STROBE checklist as Supporting Information. When completing the checklist, please use section and paragraph numbers, rather than page numbers. Please add the following statement, or similar, to the Methods: "This study is reported as per the Strengthening the Reporting of Observational Studies in Epidemiology (STROBE) guideline (S1 Checklist).”*
Thank you, we now upload a completed checklist and have included the statement in Methods (paragraph 1).

*13. Regarding reference [28] listed as ‘in press’, papers cannot be listed in the reference list until they have been accepted for publication or are otherwise publicly accessible (for example, in a preprint archive). The information may be cited in the text as a personal communication with the author if the author provides written permission to be named. Alternatively, please provide a different appropriate reference.*
Thank you, we can confirm that this paper has been accepted for publication by the Annals of Family Medicine, this is scheduled for the September 2020 issue.

*14. Where possible, please provide additional geographic, information relating to your dataset: for instance, are all practices from a small number of cities or is it an even distribution through the UK?*
Thank you, we now add (page 6): ‘CPRD GOLD is considered to be geographically and socio-demographically representative of the UK population [22].’

*15. You can omit the Data, Funding, Conflict of Interest, and Author Contributions information on page 14, as this is extracted from the data provided in the submission form.*
This change has been made, thank you.

**Reviewer #1:**
*A particular strength of this study is its employment of a diverse set of records collected from hundreds of practices, covering tens of millions of patient-years, which allowed for a retrospective analysis of the antibiotics-sepsis relationship that would otherwise have been impractical through trials. The main decision tree model is clearly structured and described, appropriate sensitivity analyses were considered, and the conclusions appear broadly valid and of interest.*
Thank you for this feedback.

*There do remain a number of possible clarifications: 1. In the "Sepsis events" section (Page 5), it is stated that "...we identified consultations for respiratory tract infections, skin infections and urinary tract infections because these are the most important groups of conditions for which antibiotics are prescribed in primary care". This however seems to imply that sepsis consultations for which antibiotic prescriptions were prescribed in the past month (i.e. for infections that weren't respiratory tract/skin/urinary tract), might have been ignored. While it is stated that these are the "most important group" of conditions for which antibiotics are prescribed, it is not clear whether they are also the most frequent group. In other words, was there a significant quantity of antibiotics prescriptions that were not covered under these three infection categories, and thus not analyzed? The supplementary data of the cited reference [25] was examined, but did not appear to cover this detail either.*

Thank you, we now acknowledge this point as a limitation on page 14, where we now say: ‘We analysed data for infection consultations in primary care and compared outcomes if antibiotics were, or were not, prescribed. However, previous studies showed that antibiotics may be prescribed at consultations with no definite diagnosis recorded.[7, 25] We did not include these prescriptions because there was no valid comparator, in terms of consultations without antibiotic prescriptions, but conclusions might have differed if missing diagnosis information had been complete.’

*2. In the "Selection of sample for antibiotic prescribing analysis" section, it is stated that a random sample of patients was drawn by selecting 10 participants for each gender/age group, also stratified by family practice. a) From our understanding, the model probabilities presented in Table 1 were obtained from this set of sampled patients. If so, this might be explicitly stated.*

Thank you for this comment. We now add explanatory notes in Table 1 (as highlighted) to show which estimates were obtained from the sample dataset and which from the entire CPRD population. The probability of an infection consultation in a 30-day period [P(Infection)] was obtained from infection consultation rates in the sample data set. The probability of an antibiotic prescription at an infection consultations [P(AB | Infection)] was estimated from the proportion of infection consultations with antibiotics prescribed in the sample dataset. All other estimates were obtained from the entire CPRD population.

*b) It is not obvious as to why the stratification by practices was required. This seems to imply that, for example, a small practice with exactly ten female patients in the 0-4 year age group for some year would have all ten of them sampled for analysis, while a large practice with say 300 such patients would then also have just 10 of them sampled, with the remaining 290 ignored.*

Thank you, we acknowledge this point on page 14, where we now say: ‘The estimates in this paper represent average values for this population of general practices and period of time…The sample design used to estimate infection consultation rates and antibiotic prescribing proportions gave each practice, and each study year, equal weight but we could have weighted the sample by practice size.’

*If this description is correct, while this might reduce certain geographic-based biases, it would nevertheless seem to omit much entirely-valid data from consideration. Moreover, even if normalization by individual family practices is desired, another option might be to compute the required probabilities with all valid patients at the practice level, and then aggregate these probabilities equally, rather than sample (and discard data from consideration) early on.*
Thank you, we now explain this more clearly on pages 6 to 7 (highlighted text), where we now say: ‘We ascertained sepsis events from the entire registered population of CPRD GOLD because these are generally rare events ...We estimated infection consultation rates and the proportion of consultations with antibiotics prescribed from a sample of patients registered with CPRD GOLD. This was because the terms of our data licence do not permit us to download and analyse data for the millions of records represented by all infection consultations and antibiotic prescriptions over 16 years [24].’

*c) Following from the above, it might be informative to have a flowchart collating the state of the data pre- and post-sampling (e.g. from the pre-sampled 66.2 million person-years to how many person-years post-sampling, from how many individual participants pre-sampling to 671,830 participants post-sampling, etc)*
Thank you, we now provide a flow-chart as Supplementary Figure 1.

*3. The degree of comprehensiveness of the EHR data might be discussed, i.e. might an individual patient visit one family practice for an infection, then visit a different practice/hospital upon onset of sepsis (not necessarily through referral)? If this is possible, would such cases be recognized/considered in the records/analysis? Also, given the relatively high mortality for sepsis (59,000 deaths from somewhat over 200,000 hospital admissions, from the Introduction), would deaths from sepsis (and possibly other complications) be expected to be recognized in the relevant records?*
Thank you, we now discuss these points on page 15, where we now say: ‘In the UK, patients register with a family practice for continuing care, but patients may utilise emergency and out-of-hours services for acute problems such as sepsis and these events may not be captured in primary care records. Providers may vary in their use of the term ‘sepsis’, which is an intermediate condition linking an infection and organ damage consequent on infection. The selection of clinical terms and medical codes is at the discretion of clinical staff, leading to lack of data standardisation. However, by using linked data we showed that inclusion of hospital episodes and mortality records did not lead to any important changes in conclusions. Further research is needed to refine, update and improve the accuracy of these initial estimates.’

*4. It is not discussed as to why infection/antibiotics prescription within the preceding 30 days was determined as the relevant period, rather than e.g. 60 days. Is 30 days a standard assumption for duration of antibiotic effect?*
Thank you for this suggestion. We have now included a new sensitivity analysis with a 60-day time window. This did not lead to any difference in interpretation. We add (page 12): ‘Analysis employing a 60-day time-window to evaluate exposure gave generally similar results to those using a 30-day time-window. In men aged 85 and over, the NNT for all infections was 262 (236 to 293) with a 30-day time-window but 313 (276 to 359) with a 60-day window; for women of the same age the figures were 385 (352 to 421) and 466 (419 to 523) respectively.’

**Reviewer #2:**

*Strengths of the study include population estimates by age, sex and frailty of this risk overall and by each infection, and provide estimates of the number of antibiotic prescriptions required to prevent one sepsis event for each of the above mentioned categories.The study uses a strong observational design and sampling method.*
Thank you for this feedback.

*There are a number of areas where the presentation and interpretation of might strengthened.First, the outcome was determined by ICD codes (presumably assigned by treating physicians or administrative staff), so there is no uniform definition of the different categories. This should be acknowledged in the limitations. Second, sepsis, urosepsis and septicemia are considered to be equivalent and are lumped together in the presentation.These conditions may vary significantly in severity, and if sensitive to the prescribed antibiotic treatment can be straightforward (septicemia and urosepsis).At a minimum, it would be very helpful to provide separate estimates for sepsis.* 
Thank you for these comments. We have now discussed these issues on page 15 where we now add: ‘Providers may vary in their use of the term ‘sepsis’, which is an intermediate condition linking an infection and organ damage consequent on infection. The selection of clinical terms and medical codes is at the discretion of clinical staff, leading to lack of data standardisation. The conditions identified as ‘sepsis’ may represent a range of disease severity, and probability estimates might be proportionately lower if only severe sepsis was included because this would reduce the apparent incidence of sepsis.’

*The two most common sources for sepsis are the lungs (pneumonia) and the kidneys. However, respiratory illnesses (including pneumonia) are often caused by viruses, although the viral infection may lead to secondary bacterial infection (e.g. influenza). Therefore, it is not surprising that the NNT is highest for respiratory infections.If there were a way to disentangle this group (perhaps presenting results for pneumonia, alone), would be useful. Similarly, it would be useful to separate UTI into cystitis and pyelonephritis.*
Thank you, we now give more detail on page 7: ‘We identified consultations for respiratory tract infections (including upper and lower respiratory tract infections), skin infections and urinary tract infections (including ‘cystitis’ and uncomplicated ‘urinary tract infections’ only) because these are the most important groups of conditions for which antibiotics are prescribed in primary care [25].’

We also acknowledge the Reviewer’s comments on page 16: ‘Respiratory tract infection consultations are extremely frequent, which may account for the lower probability of associated sepsis. Respiratory infections are often the result of virus infections and clinicians may tend to reserve the term ‘sepsis’ for bacterial infections. We evaluated uncomplicated lower urinary tract infections but estimates for the probability of sepsis might have been higher if kidney infections had been included.’

*Discussing how the authors believe their data might help antibiotics might be used more safely in greater detail would strengthen the manuscript. In this regard, it is unfortunate that the authors did not examine classes of antibiotics prescribed.One strategy for reducing emergence of antibiotic resistance is to minimize use of broad-spectrum antibiotics where possible.With additional analyses, the authors might speak more directly to this issue.*
Thank you, as noted in our reply to the Editor’s comments, our analysis cannot immediately be adapted to comparison of prescription of either ‘broad-spectrum’ or ‘narrow-spectrum’ antibiotics. We explain this on page 14: ‘We also acknowledge that in addition to changes in overall antibiotic utilisation, there have been changes in the proportion of prescriptions for broad-spectrum antibiotics. Future studies might be designed to compare the probability if sepsis if broad-spectrum or narrow-spectrum antibiotics are prescribed.’

*The justification for choosing sepsis as an outcome might be made more explicitly in the introduction and explored more thoroughly in the discussion. Appropriate antibiotic therapy has an immediate benefit that is not tied to the risk of sepsis. Sadly, the only way to limit the emergence of antibiotic resistance - which threatened our ability to treat severe infection such as sepsis - is to limit antibiotic use. It may not be reasonable to treat 30,000 individuals to prevent one case of sepsis, but there may be other reasons to do so. On the flip side - depending on the antibiotic - treating 30,000 individuals might results in 300 adverse side effects.* 

Thank you for these suggestions. We have now incorporated these into the text on page 13, where we now say: ‘Sepsis is an uncommon but concerning outcome of common infection episodes in primary care. Appropriate antibiotic therapy may have immediate benefits that are not restricted to reduction in risk of sepsis, but antibiotic prescriptions are also often associated with immediate harms in the form of drug side-effects. The potential risk of antimicrobial resistance has a significance that extends beyond the context of an individual consultation. Prescribing decisions must therefore be informed by the balance of all of the benefits and harms of either prescribing or not prescribing antibiotics. Quantification of the possible risks of sepsis contributes to informing these decisions.’


**Reviewer #3:**

*The findings of this study could have important clinical implications for practitioners that fear missing sepsis. Expressing findings in NNT is very helpful and will support understanding the clinical relevance of the findings for frontline practitioners.*

Thank you for this feedback.

*My major concern with this study concerns the reliability of these estimates given they have drawn upon data from such a large period of time. The researchers are / were up against at least three major challenges: 1. Sepsis is a tricky subject for carrying out data-linkage studies because definitions have changed multiple times over the last couple of years alone, the awareness amongst both clinicians and the public has shifted dramatically, and there have been several preventive health interventions aiming to tackle delays in the identification and management of sepsis. 2. Depending on the license agreement with CPRD, there can be limits on the number of records extracted. 3. Best practice, particularly concerning antibiotic stewardship, has changed considerably since 2002 (18 years ago). To mitigate the influence of these challenges, I am perplexed they did not endeavour to sample more patients (to the maximum of their license) from more recent years (even 2012-2017), or
at the very least in the manuscript explored this possibility +/- considered the implications of not doing so (they might have, and not had sufficient word count to do so here).*

Thank you for these comments. We have adopted the Reviewer’s suggestion. One page 9, we now explain: ‘The primary analysis reported data for a 16-year period but the incidence of recorded sepsis has been increasing.[24] We repeated the analysis using only data from 2002 to 2005 or from 2014 to 2017 to evaluate whether estimates differed from the whole period from 2002 to 2017.’ These results are now shown in Supplementary Figure 2 and Supplementary Table 5.

In the Results on page 12, we now add: ‘When the analysis was restricted to the periods from 2002 to 2005 and 2014 to 2017, estimates for the probability of sepsis were slightly higher, and NNT slightly lower for the most recent period (Supplementary Figure 2), consistent with the higher reported incidence of sepsis in this period (Supplementary Table 5). In the oldest age group from 85 years and over, the probability of sepsis without antibiotics was: 2014 to 2017, men 0.007287, women 0.004775; with antibiotics, men, 0.001290, women, 0.000839; with NNT, men 167 (141 to 202), women 254 (216 to 302).’

We also observe in the Discussion (pages 13-14): ‘The incidence of recorded sepsis has been increasing over time, with more inclusive case definitions and increasing awareness of the condition [30] [24]. When we estimated the main results for the period 2014 to 2017, the probability of sepsis was higher and number needed to treat lower than for the period from 2002 to 2017. While we caution that the absolute values of estimates may vary depending on the temporal or geographical context, we expect that in relative terms estimates will continue to identify older age, frailty and urinary tract infections as being associated with greatest risks of sepsis.’

We also caution on page 15: ‘The estimates in this paper represent average values for this population of general practices and period of time. However, we conducted a sensitivity analysis with data from 2014-2017 only.’
