# Supplementary material for: Probability of sepsis after infection consultations in primary care in the United Kingdom in 2002–2017: Population-based cohort study and decision analytic model
Source: PLoS Med. 2020 Jul 23;17(7):e1003202. doi: 10.1371/journal.pmed.1003202 (PMC7377386; doi:10.1371/journal.pmed.1003202)
Supplement: Attachment — Submitted filename: ResponseToReviews12June2020.docx [file pmed.1003202.s017.docx]

**"Probability of sepsis after infection consultations in primary care in the United Kingdom: population-based cohort study and decision analytic model" (PMEDICINE-D-20-01208R2)**

**Requests from Editors:**

*1.	Please update the title to include the study dates: “Probability of sepsis after infection consultations in primary care in the United Kingdom in 2002-17: population-based cohort study and decision analytic model”*

Thank you, the title has been changed as requested.

*2.	Please update your Competing Interests statement on the submission form to the following standard text: “The authors have declared that no competing interests exist.”*

This change has been made, thank you.

*3.	Please move the “Data sources” section from page 18 to either the Data Availability Statement in the submission form, or the Methods section of the main text, or remove it altogether.*

This change has been made, thank you.

*4.	In the Abstract, please include an additional limitation, eg. The possibility of missing or incorrect health record data, or possible sources of antibiotics outside primary care.*

Thank you, additional limitations have been mentioned in the Abstract.

*5.	Please remove the keywords from page 2. Our published articles are indexed automatically using a controlled taxonomy.*

This change has been made, thank you.

*6.	Author summary: Please spell out UTI and RTI, for clarity to non-scientist readers.*

This change has been made, thank you.

*7.	Please include line numbers in your manuscript margin.*

This change has been made, thank you.

*8.	In the section “Data source”, please provide a URL link to the database website.*

Thank you, the URL has been added.

*9.	Please cite the study protocol the same way as with other references, rather than as a hyperlink, or include the URL in brackets.*

Thank you, a URL has been added in brackets.

*10.	In the section “Selection of sample for antibiotic prescribing analysis”, please include a brief description of how the random sample was chosen, eg. by computer-generated list.*

Thank you, additional information has now been provided on page 7, line 23.

*11.	There are some instances in the results where UTI and RTI are spelled out, even though the abbreviations are already used in earlier parts of the text, eg. page 12.*

These changes have been made, thank you.

*12.	In the Discussion, please break up the long paragraph on limitations, in order to improve readability.*

This change has been made, thank you.

*13.	Thank you for addressing our comment relating to p values. Our only request is that you remove this sentence: “Readers may reflect on the substantive importance of estimated differences, and associated uncertainty intervals, for their work.”*

This change has been made, thank you.

*14.	Please correct this sentence in the Discussion to: “Future studies might be designed to compare the probability of sepsis if broad-spectrum or narrow-spectrum antibiotics are prescribed.”*

This correction has been made, thank you.

*15.	Please format your references to strict Vancouver style – bold and italics are not used.*

Thank you, the reference formatting has now been corrected.

*16.	Please correct the typo in reference 9: “Antimicrobial”*

This change has been made, thank you.

*17.	Please provide more access details (eg. A URL) for references 17, 19, and please update reference 28 to include full details rather than “in press”.*

Thank you, these changes have now been made. Reference 28 is scheduled for publication in the September issue of Annals of Family Medicine (issue 5, volume 18, 2020).

*18.	In the Discussion please replace ‘significant’ in the following sentence with a more appropriate term, eg. ‘notable’: “The lack of consistency between estimates from ecological- and individual-level analyses are likely to be explained by the significant proportion of patients…”*

This change has been made, thank you.

*19.	Please avoid the use of ‘effect’ throughout your text, given the observational nature of your study, eg. As in this sentence: “Age, gender and frailty were evaluated as effect modifiers.”*

Thank you, the word ‘effect’ has been changed to ‘association.’

*20.	The terms gender and sex are not interchangeable, please use the appropriate term.*

Thank you, the term ‘gender’ is used throughout following CPRD variable specification.

*21.	Thank you for responding to our comment 14 in the previous decision letter. To clarify, where possible, we would like you to provide a summary of sepsis events broken down by region or NHS trust, eg. as Supporting Information.*

Thank you, this information is now provided in the new Supplementary Table 3.

**Comments from Reviewers:**

*Reviewer #1: We thank the authors for considering our previous suggestions. For Supplementary Figure 1, the arrows might be labelled with brief descriptions of the selection process for convenience, or the sampling description summarized as a caption. On the additional sensitivity analyses for 2002-2005 & 2014-2017, the authors might consider including the intervening four year periods (2006-2009, 2010-2013) as well for completeness, if it is not too much trouble.*

Thank you, we have modified Supplementary Figure 1 to represent the random sampling process. We have also modified Supplementary Figure 2 to show estimates for each four- year period from 2002-2005 to 2014-2017.

*Reviewer #3: My comments have been adequately investigated and now addressed in the manuscript with appropriate discussion of their implications. The sensitivity analyses highlight the fragility of these data when different assumptions are taken. The authors have sufficiently described such limitations in the main paper, and should consider reflecting this more explicitly in the abstract.*

Thank you we have now modified the Abstract as requested.

**Production Issues:**

FIGURES and GRAPHICS:

1. *Please ensure that all main figure files are cited in ascending numerical order in the main text of the article.*

Thank you, this has been corrected.

2. *Please ensure that all main figures are referenced in the manuscript as Fig 1, Fig 2, etc. (including capitalization), rather than Figure 1, Figure 2, etc. Please note, however, that the file names themselves must not include the space, i.e. Fig1.tif, Fig2.tif, etc.*

Thank you, this has been corrected.

3. *Please ensure that all main figure legends are placed after the paragraph containing their first in-text citation.*

Thank you, this has been corrected.

TABLES:

*1. Please ensure that all main tables in their entirety (including titles and footnotes), are placed after the paragraph containing their first in-text citation.*

Thank you, this has been corrected.

*2. Tables 1 and 3 uses returns to or control text wrapping. This is indicated by a [¶ or ↵] symbol when "Show Paragraph Marks" is turned on in Word. Please either split those cells into separate cells to achieve alignment or remove the returns.*

Thank you, the ‘return’ characters have now been removed.

METADATA:

*1. Please ensure that the funders and grant numbers match between the Financial Disclosure field and the Funding Information tab in your submission form. Note that the funders must be provided in the same order in both places as well.*

Thank you, this has been corrected. We give a single funder and mention an affiliation to the Biomedical Research Centre.

*2. In accordance with PLOS' data policy, please ensure that your Data Availability Statement in the submission form clearly identifies how readers can access your data. Data cannot be available on request, or only accessible by contacting one of the authors. Note that your Data Availability Statement will be typeset as it is written, so please ensure it is in complete sentences and appears as you would like it to in the published version.*

Please see our response to point 3.

*3. In the online submission form, you indicated that "The study is based in part on data from the Clinical Practice Research Datalink obtained under license from the UK Medicines and Healthcare products Regulatory Agency. However, the interpretation and conclusions contained in this report are those of the authors alone. Requests for data access should be addressed to cprdenquiries@mhra.gov.uk . All proposals will require approval of planned use from CPRD before data release." All PLOS journals now require all data underlying the findings described in their manuscript to be freely available to other researchers, either 1. In a public repository, 2. Within the manuscript itself, or 3. Uploaded as supplementary information.*

*This policy applies to all data except where public deposition would breach compliance with the protocol approved by your research ethics board. If your data cannot be made publicly available for ethical or legal reasons (e.g., public availability would compromise patient privacy), please explain this by return email and staff will assist you with completing your Data Availability Statement.*

*At this time, we ask that you please explain, with as much detail as possible, why you are unable to make your data available in one of the three places mentioned above so we can further assess whether or not this complies with PLOS’ Data Policy and determine how we can assist you in this matter.*

Thank you for these points. As noted in our covering letter, our study was conducted by analysing data from the UK Clinical Practice Research Database (CPRD). The CPRD is a service delivered by the MHRA, which is an agency of the Department of Health (English Ministry of Health). Data access is governed by licence as outlined here: <https://cprd.com/primary-care> . The purpose of the licence is to protect patient confidentiality and ensure the integrity and security of the database. The CPRD is normally agreeable to releasing data in response to requests, but this is subject to ethical and scientific review, as is required for all CPRD studies. Incidentally, the CPRD database is widely used and there have been more than 2,000 papers published using CPRD as listed here https://www.cprd.com/bibliography . This submission follows up an email to the journal office on 9^th^ June 2020. However, in recognition of your deadline, it seemed best to resubmit the manuscript at this time. Thank you for advice on this.

ARTICLE FILE:

1. *Please indicate the corresponding author in the byline by placing an asterisk (*) after their affiliation number.*

Thank you, this has been corrected.

2. *Please include the corresponding author's email address on the title page of your manuscript, indicated by an asterisk (*). Only an asterisk and the email address itself are required.*

Thank you, this has been corrected.

*3. As your paper involves contributions from SafeAB Study Group, please format your paper as follows:*

*1) Please list SafeAB Study Group as an author in the byline.*

*2) If you wish to include the names of individual group members in the manuscript, you may list them in the Acknowledgments section.*

Thank you, this has been corrected.

*4. The name for your co-author Alastair D Hay does not match across the article file and the submission form. Please ensure that all author names match verbatim, including the use of a middle name/initials and any special characters (accents, umlauts, etc.), as correct spelling and formatting of author names is vital for accurate indexing in PubMed.*

Thank you, this has been corrected.

*5. Please ensure that your Ethics statement is available in the Methods section of your manuscript in its entirety.*

Thank you, this has been included where it says (p6) ‘The protocol was approved by the CPRD Independent Scientific Advisory Committee (ISAC protocol 18-041R).’

REFERENCES:

*1. Please ensure your references are in the style of PLOS Medicine (ICMJE/Vancouver style).*

Thank you, this has been corrected.

*2. Please put all reference citations in square brackets separated by either commas or dashes with no spaces, e.g. [1,2,3] or [1-3].*

Thank you, this has been corrected.

*3. Please make sure all references are cited in ascending numerical order in the text.*

Thank you, this has been corrected.

*4. PLOS' policy regarding references is that only published or accepted manuscripts should be included in the reference list. Papers that have been submitted but not yet accepted should not be cited. Your reference 28 is currently listed as follows: "Winter J, Charlton J, Ashworth M, Bunce C, Gulliford MC. Peritonsillar abscess and antibiotic prescribing for respiratory infection in primary care. Population-based cohort study and decision analytic model. Ann Fam Med. 2020; 18 (5) [forthcoming]"*

Thank you, we can confirm that this paper is accepted for publication.

SUPPORTING INFORMATION:

*1. Please upload your supporting information as individual files, and use the following naming format: S1 Text; S1 Fig; S1 Table, S1 Data, etc. Each file type should start as S1 and increase numerically: S1 Text, S2 Text, etc. Note that all relevant in-text citations and captions will need to be updated accordingly.*

Thank you, this has been corrected.

*2. Please ensure that all Supporting Information files use the following format verbatim throughout the manuscript (including legends and all in-text citations): S1 Fig, S1 Table, S1 Text, S1 Data, etc. Each file type should start at 1 and increase numerically. Please note, however, that the file names themselves must use an underscore rather than a space, i.e. S1_Fig.tif, S1_Table.xlsx, S1_Text.doc, S1_Data.xlsx, S1_PRISMA_Checklist.docx, etc.*

Thank you, this has been corrected.

*3. Please ensure that each supporting information file has a legend at the end of the manuscript file, after the Reference list.*

Thank you, legends have now been added as a list.

*4. Please note that your submission cannot contain any references to generic "supporting information" files and instead must refer to specific files using our naming convention (S1 Fig, S1 Table, S1 Text, S1 Data, S1 PRISMA Checklist, etc.)*

Thank you, this has been corrected.
